# A Fast and Efficient Algorithm for Mining Top-k Nodes in Complex Networks

**DOI:** 10.1038/srep43330

**Published:** 2017-02-27

**Authors:** Dong Liu, Yun Jing, Jing Zhao, Wenjun Wang, Guojie Song

**Affiliations:** 1 School of Computer and information Engineering, Henan Normal University, Xinxiang, 453007, China; 2 Engineering Technology Research Center for Computing Intelligence and Data Mining, Henan Province, Xinxiang 453007, China; 3 School of Computer Science and Technology, Tianjin University, Tianjin, 300350, China; 4 Key Laboratory of Machine Perception, Peking University, Beijing, 100871, China

## Abstract

One of the key problems in social network analysis is influence maximization, which has great significance both in theory and practical applications. Given a complex network and a positive integer k, and asks the k nodes to trigger the largest expected number of the remaining nodes. Many mature algorithms are mainly divided into propagation-based algorithms and topology- based algorithms. The propagation-based algorithms are based on optimization of influence spread process, so the influence spread of them significantly outperforms the topology-based algorithms. But these algorithms still takes days to complete on large networks. Contrary to propagation based algorithms, the topology-based algorithms are based on intuitive parameter statistics and static topology structure properties. Their running time are extremely short but the results of influence spread are unstable. In this paper, we propose a novel topology-based algorithm based on local index rank (LIR). The influence spread of our algorithm is close to the propagation-based algorithm and sometimes over them. Moreover, the running time of our algorithm is millions of times shorter than that of propagation-based algorithms. Our experimental results show that our algorithm has a good and stable performance in IC and LT model.

One company develops some new products and wants to market them. It has a limited budget so that it can only select a small group of initial users to experience the products. The company hopes that these users (top-k nodes) would like the application and to start influencing their friends to use it, and their friends would influence their friends’friends and so on, which is called the word-of-mouth phenomenon^1^,^2^,^3^. This Top-k problem, referred to as influence maximization through the powerful viral marketing. Influence maximization problem also covers a lot of other grounds in real life such as virus transmission, outbreak detection^4^ and so on. However, today’s networks turn larger and have complex connection structures, which means that the solution to the problem needs to be very efficient and accurate^5^.

In the early stage, Domingos *et al*.^2^ first study influence maximization as an algorithmic problem. Kempe *et al*. prove that the optimization problem is NP-hard^6^, present a greedy approximation algorithm applicable to all three models, which guarantees that the influence spread is within (1 − 1/*e*) of the optimal influence spread. Then, they propose a propagation-based algorithm (called general greedy algorithm). It performs well in accuracy, which wholly depends on computer operation on each round of influence estimation and it is also a continuously greedy optimizing process. That is why they runs for hours in a network that has only several thousand vertices^7^. Propagation-based algorithm significantly outperforms the other algorithms in influence spread but spends so much running time in large networks. For overcoming the drawback of propagation-based algorithm, Leskovec *et al*.^4^ present an optimization in selecting seeds, which is referred to as the Cost-Effective LazyForward (CELF). It uses the sub-modularity^8^ property of the influence maximization objective to greatly reduce the number of evaluations on the influence spread of nodes. Their experimental results demonstrate that CELF optimization could achieve as many as 700 times speedup in selecting seeds, which is a very impressive result. However, CELF still takes a few hours to complete a graph with few tens of thousands of nodes. It is still not efficient to large-scale networks. Goyal *et al*.^9^ proposed CELF++ algorithm to optimize the running time, but still has this problem in large-scale networks. Chen *et al*.^5^ proposed NewGreedy algorithm. In this propagation-based algorithm, they first removed the edges that have no impact in influencing spreading process, and then simulate influence spreading in subgraph. NewGreedy has no need to consider the estimation in each round. Its running times are 15% to 34% shorter than CELFgreeedy. Then for further improvement, Chen *et al*. proposed MixGreedy algorithm. In the first round, they use NewGreedy to select the first seed and then use the CELF optimization to select the remaining seeds in the later^5^,^10^. Notice that all the algorithms above are belong to propagation-based algorithm which needs the optimization of influence spread process.

With the rapid development of network^11^, the scale of network becomes huge^5^,^10^,^12^. The calculations of these propagation-based algorithms are facing increasing pressure.

Another kind of algorithm is topology-based algorithm. It is based on intuition attributes or experience structure and take static topology structure of network or centralities into consideration. The topology-based algorithms have no optimization of influence spread process, so the running time of them are extremely short. But the deviation degree of its influence spread can not be controlled. In previous studies, some classical indicators are considered, such as Degree heuristics^6^, Betweenness^13^ closeness^14^ and so on. K-shell decomposition^15^ and Pagerank^16^ are also a good method to measure the influence spread capability of nodes. They could fast select top-k nodes based on their intuition attributes such as Degree: a simple method that selects the k nodes with the highest degree; Distance: a simple method that selects the k vertices with the smallest average shortest-path distances to all other nodes, which is also evaluated in literature^6^. These algorithms above are all belong to topology-based algorithm.

It is worth mentioning that Chen *et al*. proposed Degreediscount^5^ which is significantly better than topology-based algorithms and is close to the influence spread of propagation-based algorithm. What’s more, the second advantage of Degreediscount is its speed, which is much faster than all propagation-based algorithms. Moreover, several excellent topology-based algorithms were proposed in recent years whose accuracy almost reach the same orders of magnitude as propagation-based algorithm. By comparison, the topology-based algorithms are getting more and more attention from researchers.

In addition, there are many excellent algorithms based on many different perspectives. Wang *et al*.^17^ proposed a community-based algorithm, which Combined the general greedy algorithm with community structure. Jung *et al*.^18^ proposed influence ranking and influence estimation (IRIE) algorithm, which has the best performance both in running time and influence spread in weighted cascade model (WC)^6^ and Trivalency model (TR)^10^. Recently, Tang *et al*.^19^ proposed an algorithm based on martingale (IMM), which performs well in continuous-time independent cascade model (CTIC)^20^. F. Altarelli *et al*.^21^ have proposed a BP-based algorithm to solve inverse problems for irreversible dynamical processes defined over networks which is based on the cavity method of statistical physics and which is capable of identifying sets of seeds which maximize some objective function that depends on the activation times of each node. Different excellent algorithms perform well in different influence propagation model. The future directions for this research change to be more pluralistic by the contribution of statistical physics, mathematical calculation, dynamical propagation and social computing.

Notice that the key point of most algorithms is to avoid “rich-club” effect^22^,^23^,^24^, which appears in social networks frequently. Degree heuristic algorithm (hereafter referred as Degree) is frequently used for selecting seeds in influence maximization but the seeds with highest degree will cause large numbers of active nodes overlapped in propagation process^23^. Briefly, the nodes with high degree often connects lots of same neighbors. Because many complex networks especially the networks with social characteristics follow a heavy-tailed distribution. In this work, we propose an algorithm based on local index rank of each vertex (LIR). The advantage of our approach is fast and efficient comparing with other algorithms. The nodes selected by LIR are the local leaders, whose degree are higher than the neighbors. In this case, LIR avoids the “rich-club” effect quickly and guarantee that most seeds are not connected to each other, which efficiently improve the Degree heuristic algorithm. We are interested in testing the performance of LIR which contains influence spread and running time. So we choose five mature algorithms from two different categories. We compared the results of our method with two excellent propagation-based algorithms and three topology-based algorithms including the best topology-based algorithm (Degreediscount algorithm). Our experimental results show that the influence spread of LIR is higher than Degreediscount and performs better than most of other algorithms in IC model. As a supplement, we simulate the influence capability in LT model. The performance of LIR in LT model is both good and stable.

## Results

### Datasets

As is shown in Table 1, N is the number of network and E is the number of edge. The average degree is *k* and the max degree of network is denoted as *k*_max_. CC refers to the average clustering coefficient of the networks and *ρ* refers to the network diameter. Ca-GrQc is a collaboration network of Arxiv General Relativity category. Each node in the network represents an author and each edge represents that two authors collaborated. Ca-Hepth is a collaboration network of Arxiv High Energy Physics Theory category from 1993 to 2003. GP2P, a sequence of snapshots of the Gnutella peer-to-peer file sharing network from August 2002. Power is an undirected, network represents the topology of the Western States Power Grid of the United States. The first three networks can be downloaded on the web at http://snap.stanford.edu/data/index.html and the last one can be downloaded on the web at http://www-personal.umich.edu/ mejn/. These four complex networks are obtained from real-life transaction. Ca-GrQc, Ca-Hepth and Gnutella follow the heavy-tailed distribution. Thus, the result curves of six algorithms in these three datasets are clear and intuitive. Notice that the max degree in Power Grid is only 19, so the influence spread of all algorithms are very close in IC and LT models. Although the importance of each nodes are hard to be distinguished, our LIR algorithm performs well in Power Grid networks.

### Experiments and Results

We summarize our experiment results in IC and LT model and calculate the running time of different algorithms. All the experiments were conducted on hardware environment with Intel(R) CoreTM i5-3470 CPU 3.20 GHz and 4 G memory and software environment with Dev C++ 5.11 on Windows 7 operating system. In our work, we use two basic models (IC and LT model) to simulate the influence spreading on real world. Mixgreedy is one of the best propagation-based algorithm both in running time and influence spread. Similarly, Degreediscount is the state-of-the-art topology-based algorithm. We use the IC model with uniform influence probability. Because different algorithms are applied to different propagation models, the accuracy and running time can not be compared to all proposed algorithms. In order to ensure the integrity of our experiments, we choose five compared algorithms which includes two excellent propagation-based algorithms and three topology-based algorithms in our experiments. In each set of experiment, we set initial seed size parameter k = 50 and take 20000 times on simulation and estimation and finally take the average of the results to obtain the influence spread^5^.

**Figure 1:**
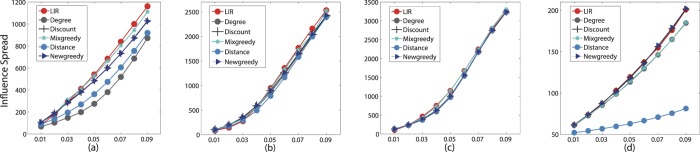
The influence spread of six algorithms on three datasets in IC model with *p* range from 00.1 to 0.09, *k* = 50. The influence spread on the y-axis against propagation probability *p* on the x-axis. (**a**) Ca-GrQc (**b**) Ca-Hepth (**c**) GP2P (**d**) Power.

**Figure 2:**
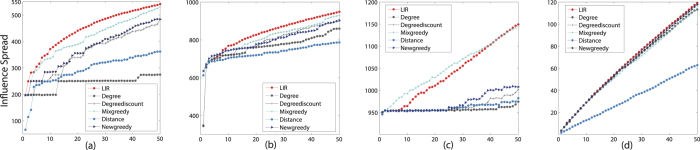
The influence spread of six algorithms on three datasets in IC model with *p* = 0.05, *k* ranges from 1 to 50. The influence spread on the y-axis against parameter *k* on the x-axis. (**a**) Ca-GrQc (**b**) Ca-Hepth (**c**) GP2P (**d**) Power.

First, we test the six algorithms in IC model. In literature^6^, they mainly report results on a small propagation probability of *p* = 0.01, but we also consider other values (*p* = 0.02 − *p* = 0.09) and add the experiments correspondingly. Notice that larger p values (e.g. *p* = 0.1) are not considered due to its insensitivity to different algorithms^6^. When the propagation probability *p* exceeds 0.1, the difference of influence spread almost disappeared. For testing the sensitiveness of propagation probability p, we shows the results of experiments of six algorithms in Fig. 1, which depict the variety of influence spread with p increasing (0.01 to 0.09). In Fig. 1, it is easy to find that our LIR algorithm performs better than other algorithms. Especially in Fig. 1(a), from *p* = 0.04 to *p* = 0.09 the influence spread of LIR surprisingly exceed all algorithms. The similar phenomenon also comes up in Fig. 1(b) when *p* > 0.04. In Fig. 1(c), LIR gets ahead of the rest algorithms from *p* = 0.03 to *p* = 0.07. When the propagation probability is low (e.g. *p* < 0.4), the influence spread of LIR and other algorithms are very close. In Fig. 1(d), Power Grid networks which max degree is very small, our LIR still performs better than Mixgreedy algorithm and has no big gap with Newgreedy and Degreediscount algorithms. The influence spread results are the average of the 20000 experiments, so the numerical difference of the six algorithms is not obvious. For example, in Fig. 1(b,c), the network size are large whereas the influence spread result of our LIR algorithm exceeds other algorithms within 100 nodes, which cause the overlapping of carves. In summary, our LIR algorithm as a topology-based algorithm performs better than other algorithms in IC model. For showing the details of the results clearly, we draw Fig. 2 which with the median of all propagation probability (i.e. *p* = 0.05).

Figure 2 reports the results on influence spreads of our algorithms as well as other algorithms for comparison. The difference between Fig. 1 is that the x-axis represents the parameter k (from 1 to 50). As Fig. 2 shows, the influence spread of various algorithms on the four graphs in the IC model with *p* = 0.05, which are very consistent. It is easy to read that the red dots are our LIR algorithm. Distance and Degree heuristics are significantly worse than the other algorithms. LIR is an improved method based on Degree. The influence spread of LIR is much higher than Degree except in Power gird network. In Fig. 2(a–c), the influence spread of LIR is higher than the other algorithms, even Mixgreedy and Newgreedy are lower than it. Notice that the influence spread of Degreediscount is lower than LIR in these four datasets all the time. Compared to Degree and Distance heuristics, LIR avoid the “Rich-club Effect” subtlety, so the influence spread of LIR must be higher than the other three topology-based algorithms.

Table 2 reports the standard deviation of different algorithms for selecting *k* = 50 seeds in IC model with *p* = 0.05. For each algorithm, we simulate 20000 times of influence spread and compute the standard deviation. As Table 2 shown, the gap between LIR and other mature algorithms is not large, which means that the rationality of LIR is proved.

Table 3 reports the running times of six algorithms. Our LIR algorithm only take *O(m*) time in a network, so the liner running time of LIR is amazingly short. It is obvious that LIR and Degreediscount run extremely fast, only take a few milliseconds to finish, and are nearly hundred-thousand times faster than the propagation-based algorithms. Although LIR spends a little more time than Degreediscount in the first three networks, the influence spread of LIR is higher than Degreediscount in the three networks. In GP2P, LIR is better than Degreediscount both in running time and influence spread. Generally, LIR take merely the same time as Degreediscount but has a higher influence spread than Degreediscount. The running time of Distance is almost same with the propagation-based algorithms while its influence spread is much worse than the propagation-based algorithms. Because LIR is a topology-based algorithm, the running time of it is not related to the influence model. Thus, we only show the time statistics in IC model in this work.

**Figure 3:**
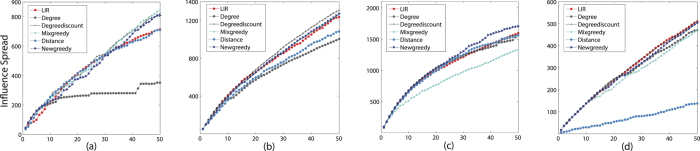
The influence spread of six algorithms on four datasets in LT model k = 50. The influence spread on the y-axis against parameter *k* on the x-axis. (**a**) Ca-GrQc (**b**) Ca-Th (**c**) GP2P (**d**) Power.

Figure 3 shows the influence spread of six algorithms on four datasets in LT model. The two propagation-based algorithms are not a surprise that it generates the best influence spread. The performance of LIR is not as good as the result in IC model. However, the influence spread of LIR essentially match the propagation-based algorithms. In Fig. 3(a), Degree heuristic performs worst, but the two propagation-based algorithms have a good performance. In Fig. 3(b), the influence spread of LIR is close to the two propagation-based algorithms and Degreediscount. In Fig. 3(c), LIR’s influence spread is higher than Mixgreedy and the influence spread of LIR is higher than Degreediscount and Mixgreedy. In Fig. 3(d), the accuracy of LIR and Degreediscount are almost same even higher than the other propagation-based algorithms.

In summary, our LIR runs fast and obtains a higher influence spread, which is generally close to propagation-based algorithms or exceeds the two propagation-based algorithms sometimes. Moreover, the running time of LIR is million times shorter than propagation-based algorithms. As for Degree heuristic, LIR is an excellent improved method, which avoid rich club effect successfully. Degreediscount performs well both in running time and influence spread accuracy. LIR takes almost the same running time as Degreediscount but gets a higher influence spread in IC model. Therefore LIR is a suitable choice for the influence maximization problem.

## Discussion

Degree algorithm selects the seeds that with highest degree. However, in most real life network, the degree of nodes follows a heavy-tail distribution, which indicates that most nodes with high degree are connected with each other (rich-club phenomenon). So, only considering degree of nodes will cause the overlapping in the first step of influence spread. From this point of view, LIR solve the rich-club problem well and reduce the number of overlapped neighbors. Nodes with 0 *LI* value mean that they are the leader in local part and most of them are not linking to each others.

**Figure 4:**
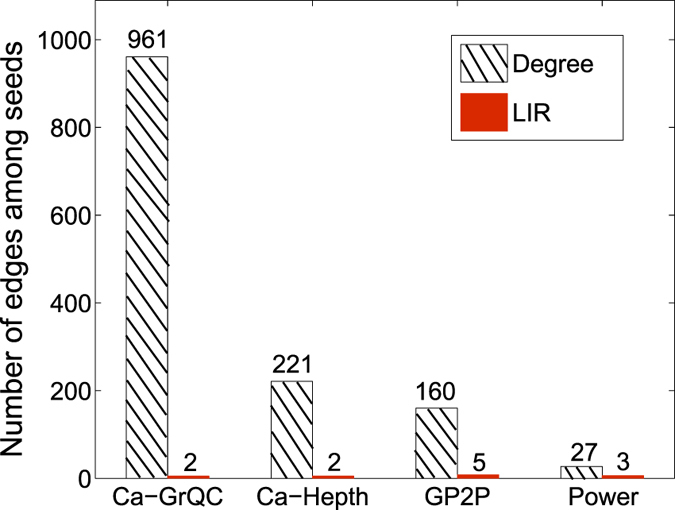
The number of edges that connect two seeds in the seed set (50 seeds) selected by LIR and Degree algorithms.

In Fig. 4, we show the number of edges that connect two seeds in the seed set selected by LIR and Degree algorithms. There are 961 edges among the seeds selected by Degree algorithm in Ca-GrQC, but only 2 edges among the seeds selected by our LIR algorithm. In other three datasets, the seeds selected by LIR are merely connected. In the four datasets, the edges among the seeds selected by LIR are very sparse. The probabilities of selecting two connected nodes by our method are lower than 0.5%. So it is obvious that LIR largely improves the Degree algorithm, and the combined influence spread ability of these dispersed seeds will be stronger.

Notice that the greater gap between two algorithms in the Fig. 4, the better LIR performs in influence spread. For example, in Ca-GrQC, LIR only has 2 edge connect seeds but Degree algorithm has 961 edges, which is a big difference. So in Fig. 2(a), Degree is the worst algorithm compared with other algorithms but LIR is the best. On the contrary, in Power network, the max degree is not high and many nodes have same degree, which makes our method insensitive. The difference between our algorithm (4 edges) and Degree (27 edges) is not large. So in Fig. 2(d), the performance of LIR and other algorithms are very close. Thus it can be seen that avoiding the rich-club is meaningful in identifying top-k influential nodes. We also assume that this idea is from the origin of the communities characteristic, which indicates the top-k nodes we finally select may located in different communities or at least there has a certain distance between each others^14^,^17^,^25^.

Several conclusions can be drawn from the experimental results reported above. First, comparing with original Degree algorithm, LIR is a well-performed improved method. Second, the liner running time of LIR is in same order of magnitude as Degreediscount while showing very impressive results in the independent cascade model. Last but not least, the influence spread of LIR match the propagation-based algorithms and even exceeds them.

To summarize, our LIR is a fantastic new approach for solving the influence maximization problem, which only takes less than one-millionth of time of propagation-based algorithms but performs considerably well in influence spread. There are also several plans for this research. First, our LIR performs well in the independent cascade model. We plan to optimize LIR to make it adapt to other propagation models and try to find a strategy to ensure all leaders are not connect to each others. Second, we will analysis poor performing results in LT model and find the reason. Third, we will continue to change the probability of IC model and explore the relevance of different probability and influence spread. We also plan to investigate the top-k problem based on the view of community structures in our future work.

## Methods

### LIR algorithm

*G* = {*V, E*} is a network or graph where *V* is set of vertices (or nodes) and *E* ⊆ *V* × *V* is a set of edges (or links between the nodes). *d*_*i*_ is the degree of node *v*_*i*_. *N(v*_*i*_) = {*v*_*j*_|*v*_*j*_ ∈ *V*,(*v*_*j*_, *v*_*i*_) ∈ *E*} that is the neighborhood set of node *v*_*i*_. We define that 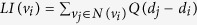. *Q(x*) = 1 when (*x* > 0), otherwise *Q(x*) = 0. We compute the *LI* of each node first, which represents the local index of each node. Normally, if the *LI* value of node *v*_*i*_ is 5, it indicates that there are 5 neighborhoods around node *v*_*i*_ whose degree are higher than node *v*_*i*_. Notice that according to the definition of LI, it is possible that two adjacent nodes have the same *LI* value. (e.g. two connected nodes that have the highest degree in the network). In this case, there will be two nodes whose *LI* value are 0 simultaneously. But if the networks follow heavy-tailed distribution, this phenomenon will happen with extreme low probability, which has merely no impact on our experimental results. In this paper, We are interested in the nodes with 0 *LI* value. These 0-*LI* nodes have two advantages: one is that they all have the highest degree in their local part, the other is that most of them are not adjacent to each others. Finally, we rank these 0-*LI* nodes by their degree. The length of LIR is depended on *k*. Algorithm 1 as the following implements the steps of LIR.


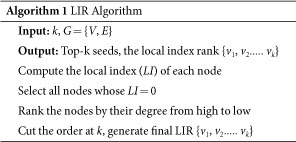


### Illustrate examples

LIR is an improved approach based on Degree heuristic, which inherited the advantages of short running time. Degree heuristic is an intuitive method for the influence maximization problem^14^,^26^, but its fatal flaw is that it can’t deal with the “rich club” effect, which caused a large number of nodes overlapped in spread process. Most topology-based algorithms focus on finding a way to avoid the “rich-club” effect. The approach that selects 0-*LI* nodes solves the problem easily and fast. LIR is an ordered set by degree from high to low and the *LI* value of all the seeds we select are 0. In this way, LIR can obtain more neighbors of seed set in the first step of influence spread, which could make the influence spread larger.

**Figure 5:**
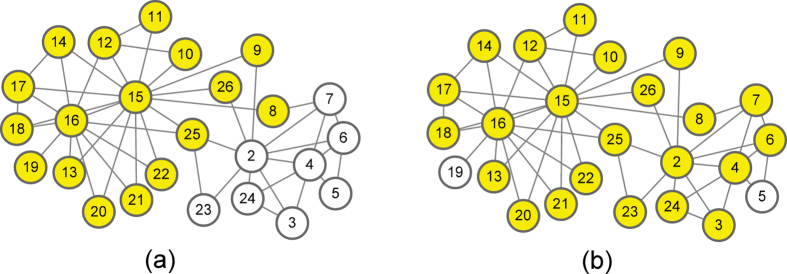
Two steps of sampling nodes based on expansion contribution in a simple network. Nodes with yellow color are the neighbors of seeds (include seeds).

We display a sample network in Fig. 5 to demonstrate the difference between LIR and Degree. Nodes with yellow color are the neighbors of seeds (include seeds). Suppose that we want to choose two seeds in this network and get more nodes infected by the seeds. If we use Degree-based algorithm, we should find the nodes with high degree in the network. In this way, node 15(degree is 15) and 16(degree is 11) will be selected. Notice that seeds 15 and 16 leave 8 nodes (white color nodes) that have no chance to be active in the first step of influence spreading. Although the degree of node 15 and 16 are high, most of their neighbors are overlapped. In Fig. 5(b), Because node 15 and 16 are connected, the LI value of 16 is 1(not 0). The degree of node 2(degree is 9) is not high, but its LI value is 0. Then, we choose node 15 and 2 as seeds based on LIR algorithm, whose *LI* value is 0, the most nodes in the network turn to be the seeds’neighbors. It is obvious in Fig. 5(b) that only 2 nodes leave which have no chance to be active. In general, using LIR to choose seeds could make seeds more dispersed and then bring more neighbors.

### IC Model

IC (independent cascade model)^6^. Given a network *G* = {*V, E*}, where the vertex set *V* has *n* nodes. There exists an edge *e(uv*) between vertex *u* and its neighbor *v. p*_*uv*_ ∈ [0, 1] is the probability that *u* infect *v*, which is independent in the whole diffusion process. If at time *t, u* is active and its neighbor *v* is not yet activated, *u* try to infect *v* with probability *p*_*uv*_. If this process is successful, *v* will be active in *t* + 1 time. But whether it succeeds or not, *u* has only one chance to infect *v*. If *v* has many neighbors in time, the order of the neighbors try to infect *v* is arbitrary. The simulation process starts from the initial node, until no new node can be activated. In IC model with large or small propagation probability *p*, the influence spread is not very sensitive to different algorithms. We had test the probability 0.01 to 0.1. In order to show the curves clearly, we added Fig. 2 with the median *p* = 0.05.

### LT Model

LT (liner threshold model)^6^. Given a network *G* = {*V, E*}, *N(u*) is the set of neighbors of *u*. Suppose *u* is active, *u* has an influential value on its neighbor, denoted as *b*_*uv*_. The *b*_*uv*_ on edges could be learned from real data. In our experiment, we assign equal values to edges. For example, if a node has 10 neighbors, then *b*_*uv*_ of each edge is 0.1. All nodes in *N(u*) have influential value to *u*, but the summation is less than 1. That is ∑_*v*∈*N(u*)_*b*_*uv*_ ≤ 1. Each threshold of *u* has to be generated uniformly at random from 0 to 1. (i.e. with stochastic threshold, with random distribution), if ∑_*v*∈*N(u*)_*b*_*uv*_ ≥ *θ*_*u*_, *u* will be active. In LT model, an active node *u* failed to infect his neighbor *v*, its influential value *b*_*uv*_ will be accumulated, which is a contribution to the other neighbors. The diffusion process is over until no new node can be activated.

## Additional Information

**How to cite this article**: Liu, D. *et al*. A Fast and Efficient Algorithm for Mining Top-k Nodes in Complex Networks. *Sci. Rep.* **7**, 43330; doi: 10.1038/srep43330 (2017).

**Publisher's note:** Springer Nature remains neutral with regard to jurisdictional claims in published maps and institutional affiliations.

## Figures and Tables

**Table 1:** Specific statistical parameters of the real datasets.

Name	*N*	*E*	*k*	*k*_max_	*CC*	*ρ*	type
Ca-GrQc	5242	14496	5.5	81	0.5296	17	undirected
Ca-Hepth	9877	25998	5.2	65	0.4717	17	undirected
GP2P	8114	26013	6.4	55	0.0095	10	directed
Power	4941	6594	2.7	19	0.08	19	undirected

**Table 2:** The standard deviation of 20000 times influence propagation simulation on four datasets in IC model, p = 0.05, k = 50.

Standard Deviation	Ca-GrQc	NetHEP	GP2P	Power
LIR	37.2114	67.0028	77.0103	11.4147
Degree	42.3721	76.1460	88.0991	10.7248
Degreedisocunt	33.7725	65.1861	85.4825	11.4707
Mixgreedy	40.5072	64.2908	78.1165	10.7426
Distance	43.7913	74.016	87.2103	7.3039
Newgreedy	33.0334	65.192	84.4017	11.4003

**Table 3:** The running time of six algorithms on four datasets in IC model, p = 0.05, k = 50.

Running time (s)	Ca-GrQc	NetHEP	GP2P	Power
LIR	0.00626	0.00622	0.00326	0.00718
Degree	0.00154	0.00307	0.00151	0.00308
Degreedisocunt	0.00263	0.00366	0.00382	0.00342
Mixgreedy	1059.41	1084.48	4611.93	57.099
Distance	394.797	3120.40	2104.55	526.83
Newgreedy	855.926	1767.78	1557.65	985.928
